# 一个复合杂合突变导致的遗传性凝血因子Ⅺ缺陷症家系

**DOI:** 10.3760/cma.j.issn.0253-2727.2021.08.014

**Published:** 2021-08

**Authors:** 晓勇 郑, 艳慧 金, 瑶瑶 徐, 丽红 杨, 丽青 朱, 欢欢 王, 淑婷 蒋, 明山 王

**Affiliations:** 温州医科大学附属第一医院医学检验中心，温州 325015 The First Affiliated Hospital of Wenzhou Medical University, Wenzhou 325015, China

遗传性凝血因子Ⅺ（FⅪ）缺陷症是一种罕见的常染色体遗传病，全球年发病率约为百万分之一，在德系犹太人中高达1/450[1]。其主要临床表现为创伤或手术后出血难止，尤其是在口腔、泌尿道等高纤溶部位较为多见，自发性出血少见，无性别差异[2]–[3]。本文报告一个遗传性FⅪ缺陷症家系凝血表型和F11基因检测结果并初步探讨其分子致病机制。

## 对象与方法

1. 家系资料：先证者，女，44岁，既往体健，肝肾功能正常，因突发性耳聋拟行溶栓治疗，查凝血功能指标显示活化部分凝血活酶时间（APTT）为93.9 s（参考值29.0～43.0 s）且能被正常血浆纠正，FⅪ活性（FⅪ∶C）2％，FⅪ抗原（FⅪ∶Ag）4.5％，其他凝血功能指标均正常。其家系成员（共3代5人）均无出血及血栓病史，先证者父母非近亲婚配。家系遗传图见图1。

**图1:**
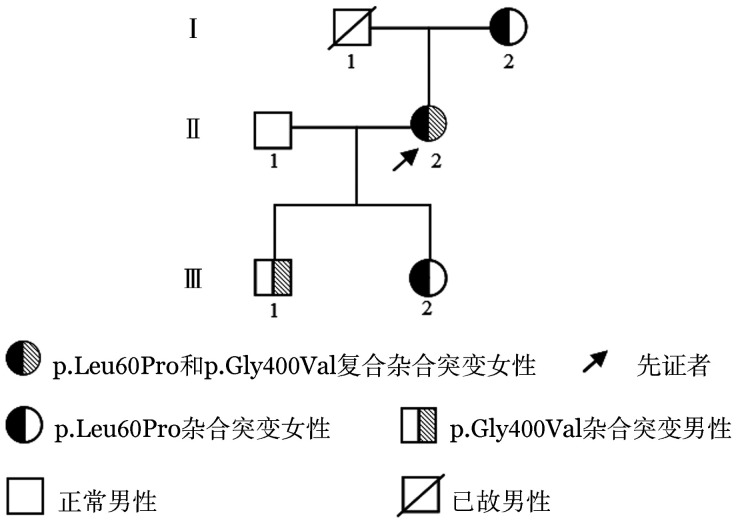
凝血因子Ⅺ缺陷症家系图

2. 健康对照组：正常对照为100名我院健康体检者，以建立本实验室凝血指标生物参考值及用于排除基因多态性。男53名，女47名，年龄22～56岁，均无肝、肾功能疾病，且无其他基础性疾病。所有受试者均知情同意。

3. 标本采集和处理：采集所有受试者外周血2.7 ml，109 mmol/L枸橼酸钠1∶9抗凝，分离乏血小板血浆、血细胞，分别用于临床表型检测、基因组DNA提取。

4. 实验室凝血指标检测：采用Stago-STA-R全自动血凝仪（法国Stago公司产品及配套试剂）一期凝固法检测凝血酶原时间（PT）、APTT、纤维蛋白原（FIB）、凝血酶时间（TT）、凝血因子Ⅷ活性（FⅧ∶C）、凝血因子Ⅸ活性（FⅨ∶C）、FⅪ∶C、凝血因子Ⅻ活性（FⅫ∶C）和狼疮抗凝物。免疫比浊法检测D-二聚体（D-D）。采用ELISA法测定FⅪ∶Ag。

5. 全血基因组DNA提取及PCR扩增：使用北京天根生化科技有限公司提供血液样本基因组DNA提取试剂盒提取全血基因组DNA。PCR引物由上海桑尼生物科技有限公司合成，引物序列参见文献[4]。PCR反应体系25 µl，其中Taq PCR Mastermix 12.5 µl，DNA模板2 µl，正向引物1 µl，反向引物1 µl，ddH^2^O 8.5 µl。PCR反应步骤：95 °C预变性5 min，95 °C变性30 s，55 °C～ 60 °C退火30 s，72 °C延伸30 s；扩增30个循环后，72 °C延伸10 min。扩增结束后，取PCR产物5 µl经15 g/L琼脂糖凝胶电泳鉴定，用GoldviewⅠ标记PCR产物大小。

6. F11基因测序：将PCR扩增产物送上海桑尼生物工程有限公司进行测序。用Chromas软件将测序结果与美国NCBI基因库公布的F11基因序列（GenBank AY191837）比对，寻找突变位点，再通过反向测序进行验证。待明确突变位点后，再做其家系成员相应位点的PCR扩增及测序。

7. 生物信息学特性分析：用ClustalX-2.1-win软件将人类FⅪ突变氨基酸与其同源物种小家鼠（Mus musculus）、黑猩猩（Pan troglodytes）、家犬（Canis lupus familiaris）、牛（Bos Taurus）和西方爪蟾（Xenopus tropicalis）（同源物种氨基酸序列来源：https://www.ncbi.nlm.nih.gov/homologene）的氨基酸序列进行比对，分析氨基酸的保守性；采用MutationTaster（http://www.mutationtaster.org）、PolyPhen-2（http://genetics.bwh.harvard.edu/pph2/index.shtml）、PROVEAN和SIFT（http://provean.jcvi.org/index.php）评估突变氨基酸对蛋白功能的影响（蛋白质参考ID P03951，蛋白质参考转录本ID ENST00000403665）；使用Swiss-PdbViewer4.0.1软件分析FⅪ蛋白模型突变前后氨基酸结构和次级键的改变（FⅪ蛋白模型文件pdb：2f83）。

## 结果

1. 先证者及家系成员凝血指标结果：先证者APTT延长为93.9 s，FⅪ∶C和FⅪ∶Ag分别为2％和4.5％；先证者母亲、儿子、女儿APTT稍延长，FⅪ∶C和FⅪ∶Ag明显降低，先证者及家系成员的其他凝血指标均在正常参考范围内，详见表1。

**表1:** 先证者及家系成员凝血表型指标结果

家系成员	PT（s）	APTT（s）	FIB（g/L）	TT（s）	D-D（mg/L）	FⅧ∶C（％）	FⅨ∶C（％）	FⅫ∶C（％）	FⅪ∶C（％）	FⅪ∶Ag（％）
先证者（Ⅱ_2_）	14.1	93.9	3.86	14.2	0.23	98	96	97	2	4.5
母亲（Ⅰ_2_）	13.6	48.2	3.45	16.7	0.14	110	104	106	46	55.0
丈夫（Ⅱ_1_）	13.1	34.4	3.29	18.2	0.11	120	110	112	105	101.0
儿子（Ⅲ_1_）	13.9	51.6	2.06	15.2	0.20	114	101	103	32	43.0
女儿（Ⅲ_2_）	14.3	53.3	2.27	16.1	0.16	106	99	101	31	41.0

参考值	11.6～14.6	29.0～43.0	2.00～4.00	14.0～20.0	<0.50	86～125	86～114	82～118	82～118	90.0～110.0

注：PT：凝血酶原时间；TT：凝血酶时间；APTT：活化部分凝血活酶时间；FIB：纤维蛋白原；D-D：D-二聚体；FⅪ∶Ag：凝血因子Ⅺ抗原；FⅧ∶C、FⅨ∶C、FⅪ∶C、FⅫ∶C分别为凝血因子Ⅷ、Ⅸ、Ⅺ、Ⅻ活性

2. F11基因测序结果：F11基因分析发现先证者存在2个突变基因位点：位于4号外显子上的c.233T>C（p.Leu60Pro）杂合错义突变和11号外显子上的c.1253G>T（p.Gly400Val）杂合错义突变。母亲和女儿检出p.Leu60Pro杂合子，儿子检出p.Gly400Val杂合子，丈夫为野生型（图2）。p.Leu60Pro突变在100名健康体检者中未检出，从而排除了基因多态性。查阅国内外文献及相关网站，未见p.Leu60Pro突变位点的报道。

**图2:**
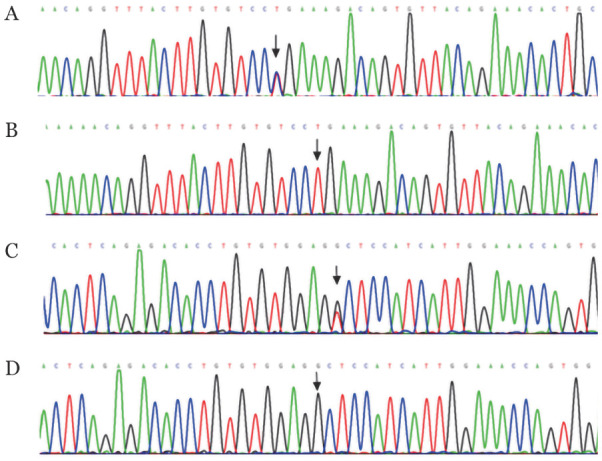
F11基因第4号、11号外显子测序图 A：c.233T>C杂合突变型；B：c.233T>C野生型；C：c.1253G>T杂合突变型；D：c.1253G>T野生型

3. 突变氨基酸保守性分析：用ClustalX-2.1-win软件对人类和5个同源物种（黑猩猩、家犬、牛、小家鼠、西方爪蟾）的F11基因氨基酸序列进行多重比对，结果显示Leu60在物种间保持高度保守（图3）。

**图3:**
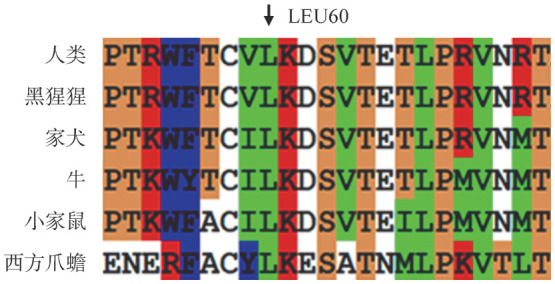
F11基因Leu60位点保守性分析结果

4. 突变蛋白功能预测评分：四个在线生物信息软件MutationTaster为1.00分、PolyPhen-2为1.000分、PROVEAN为−3.04分、SIFT为0.001分，均显示p.Leu60Pro突变为有害的，可能影响蛋白质的功能，有一定致病性。

5. 蛋白模型分析：分析p.Leu60Pro突变前后模型，在野生型中，Leu60主链与Ile17主链、Thr18主链各形成一条氢键，当Leu60突变为Pro60时，其氢键没有改变，但增加了一个苯环，使蛋白质的结构发生了改变。见图4。

**图4:**
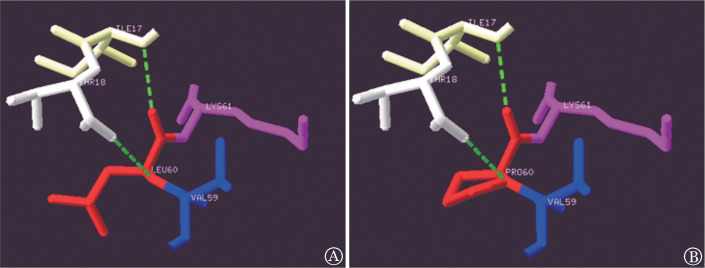
p.Leu60Pro突变型及野生型蛋白质模型分析图 A：野生型；B：突变型。绿色虚线代表氢键

## 讨论

FⅪ主要是以丝氨酸蛋白酶原形式存在于血液中，与高分子量激肽原（HMWK）以非共价键结合形成复合物[5]，总分子量160×10^3^。FⅪ由两个相同的包含607个氨基酸的单体亚基组成，在Cys321通过二硫键连接形成同源二聚体结构，每个亚基包含4个AP域：AP1结合凝血酶，AP2结合HMWK，AP3结合凝血因子Ⅸ（FⅨ）、血小板膜糖蛋白Ⅰb（GPⅠb）和肝素，AP4是两个FⅪ单体在Cys321通过二硫键结合形成二聚体的部位[6]–[7]。

本例先证者APTT明显延长（93.9 s），FⅪ∶C和FⅪ∶Ag均明显降低，诊断为Ⅰ型遗传性FⅪ缺陷症。基因分析发现先证者存在F11基因4号外显子p.Leu60Pro杂合错义突变和11号外显子p.Gly400Val杂合错义突变，母亲和女儿存在p.Leu60Pro杂合错义突变，儿子存在p.Gly400Val杂合错义突变。根据遗传家系图可见p.Leu60Pro突变来源于母亲，p.Gly400Val应该来源于先证者已故的父亲。Gly400位于FⅪ高度保守的组氨酸环催化结构域附近，被Val取代后，可能改变了蛋白质结构和功能。有研究显示，在转染的细胞培养基中几乎检测不到p.Gly400Val的FⅪ蛋白质，并且在p.Gly400Val的细胞内F Ⅺ蛋白质水平较野生型降低22.2％～53.5％[8]，这表明该蛋白的降解速度比野生型FⅪ更快，该突变是导致FⅪ水平降低的原因之一。

Leu60位于AP1的裸露环中，参与形成复杂的弯曲反向平行的β-折叠。同源性分析显示Leu60在6种物种间保持高度保守，表明该位点在蛋白中起到重要的作用。四个在线生物信息软件均显示p.Leu60Pro突变为有害突变，可能会影响FⅪ蛋白的结构和功能。有研究显示，同在AP1结构域内的p.Thr33Pro突变不会影响FⅪ蛋白合成，但会导致FⅪ蛋白分泌减少，可能是由于α-螺旋结构的破坏引起AP1结构域折叠的改变[9]–[10]。p.Leu60Pro突变可能也会有与p.Thr33Pro突变相似的分子致病机理。本研究氨基酸突变模型分析显示，Leu60主链与Ile17、Thr18主链之间各自存在一条氢键，当亮氨酸被脯氨酸取代后，其氢键并没有改变，但侧链增加了一个苯环。这种从中等大小的中性氨基酸到带苯环的酸性氨基酸变化产生了位阻效应，可能影响蛋白质局部构象和静电的改变。

综上，p.Gly400Val和p.Leu60Pro复合杂合错义突变是导致本家系先证者FⅪ降低的主要原因，p.Leu60Pro杂合错义突变为鲜见报道的基因突变位点，但其确切分子致病机制有待进一步研究。
